# Targeting Myostatin as an Adjunct Treatment for the Preservation of Cardiometabolic and Skeletal Muscle Function in Type 1 Diabetes

**DOI:** 10.3390/ijms26104830

**Published:** 2025-05-18

**Authors:** Emily Nunan, Denton R. Huff, Jillian L. Gore, Carson L. Wright, Tag Harris, Landon Butler, Caleb A. Padgett, Matthew T. Rochowski, Pamela C. Lovern, Ali Boolani, Cammi Valdez, Joshua T. Butcher

**Affiliations:** 1 Department of Physiological Sciences, College of Veterinary Medicine, Oklahoma State University, Stillwater, OK 74078, USA; 2 Vascular Biology Center, Medical College of Georgia, Augusta University, Augusta, GA 30912, USA; 3 Human Performance and Nutrition Research Institute, Oklahoma State University, Stillwater, OK 74078, USA; ali.boolani@okstate.edu; 4 Department of Physical Sciences, Northeastern State University, Tahlequah, OK 74464, USA; valdez07@nsuok.edu

**Keywords:** type 1 diabetes, myostatin, glucose homeostasis, skeletal muscle, endothelium, metabolism, muscle performance

## Abstract

Type 1 Diabetes Mellitus (T1D) is a disease characterized by the destruction of pancreatic beta cells. The subsequent loss of insulin production results in hyperglycemia, muscle wasting, and vascular dysfunction. Due to an inability to appropriately maintain glucose homeostasis, patients afflicted with T1D suffer from increased morbidity and early mortality. Skeletal muscle is the body’s largest metabolic reservoir, absorbing significant amounts of glucose from the bloodstream and physical exercise is known to improve and prevent the progression of pathological outcomes, but many T1D patients are unable to exercise at a level that conveys benefit. Thus, directly targeting muscle mass and function may prove beneficial for improving T1D patient outcomes, independent of exercise. A potent negative regulator of skeletal muscle has been identified as being upregulated in T1D patients, namely the myokine myostatin. Our hypothesis is that targeting myostatin (via genetic deletion) will prevent glucose dysfunction in a T1D model, preserve skeletal muscle function, and protect against vascular and renal dysfunction. Our methods utilized adult male mice with (WT) and without myostatin (Myo KO), in combination with the chemical induction of T1D (streptozotocin). Experimental outcomes included the assessment of glucose homeostasis (plasma glucose, HbA1c, IGTT), metabolism, muscle function (in vivo plantarflexion), and skeletal muscle vascular function (ex vivo pressure myography). Our results described systemic benefits from myostatin deletion in the T1D model, independent of insulin, including the following: inhibition of T1D-induced increases in plasma glucose, prevention of functional deficits in muscle performance, and preservation of fluid dynamics. Further, endothelial function was preserved with myostatin deletion. Taken together, these data inform upon the use of myostatin inhibition as a therapeutic target for effective treatment and management of the cardiometabolic and skeletal muscle dysfunction that occurs with T1D.

## 1. Introduction

Diabetes Mellitus (DM) is characterized by the body’s inability to adequately control blood glucose levels. Broadly speaking, there are two subclasses of DM that have been characterized by either the inability of the body to produce insulin (Type 1) or the progressive development of insulin resistance in tissues (Type 2). Regardless of the type of diabetes, the result is an inability of tissues to access the circulating pool of energy residing within carbohydrates that classically drives aerobic oxidation, and thus the insulin-sensitive tissues (such as skeletal muscle) experience starvation. The clinical signs of this disease normally include polyuria (excessive urination), polydipsia (excessive drinking), polyphagia (insatiable hunger), weight loss/muscle wasting, and lethargy. Parameters for diagnosis according to the American Diabetes Association are a fasting blood glucose level greater than 126 mg/dL (hyperglycemia) and a glycosylated hemoglobin percentage (HbA1C) over 6.5% in humans. Additional diagnostic assessments include an oral glucose tolerance test for the determination of glucose clearance, with a blood glucose level over 200 mg/dL after two hours indicating the presence of the disease [1].

This study focused on Type 1 Diabetes Mellitus (T1D), which has classically been referred to as juvenile diabetes due to the average age of diagnosis typically occurring in children and teens. However, the increasing heterogeneity observed in the ages of the population diagnosed with T1D, particularly adult-onset, suggests that juvenile diabetes may be a misnomer [2,3,4]. Currently, T1D does not have a known trigger, but the result is an undetermined autoimmune disorder that targets and destroys pancreatic beta cells. As pancreatic beta cells are the only described cell able to produce insulin, their permanent destruction renders this disease irreversible. While only a small percentage of the diabetic population in the U.S. are diagnosed as T1D (5.7%), it is a chronic life-long disease [5]. Despite advances in early diagnosis and treatment, a TID patient will face a reduced lifespan (lessened by 10–17 years) due to increased cardiovascular risk factors [6,7,8]. As such, disease management is overwhelmingly focused on the maintenance of glucose homeostasis and prevention of hyperglycemia, which have been enabled with advances in continuous glucose monitoring and insulin pumps [1,9]. However, there remain significant health and economic barriers in treating the disease and, although the organ-specific drivers of pathophysiology are still being explored, hyperglycemia is the main correlate and remains seen as the driving factor of the systemic complications of T1D [10,11,12].

As mentioned previously, the lack of insulin production results in dangerously excessive blood glucose levels in T1D patients. Pathological complications arise from macro- and microvascular damage caused by increased levels of glucose in the blood, and include retinopathy, nephropathy, neuropathy, myopathy, and cardiovascular disease—typically thickening the arteries and arterioles [13,14,15]. The microvascular damage also leads to endothelial dysfunction that has been shown to be a significant prognostic factor for developing hypertension [16,17]. However, even with proper glycemic control, studies have shown that T1D patients develop a positively correlated insulin resistance with the duration of the disease and decreased lifespan [18]. Insulin resistance, defined as the increasing insensitivity of tissues towards insulin, is well associated with type 2 diabetes, but is also a critical factor in T1D. Hyperglycemia causes an increase in advanced glycation end products that contribute to inflammatory and oxidative stress pathways, but also interfere with insulin signaling [19]. Proper glycemic control or a shift towards normoglycemia does reduce or resolve the complications, especially the cardiovascular effects, of T1D [10].

Regular physical exercise has been known to be associated with multiple health benefits such as improvements in cardiovascular function, protection from morbidities such as kidney injury, obesity, and metabolic dysfunction, and improved quality and quantity of muscle mass [20]. Another effect of exercise is an increase in insulin sensitivity and protection from hyperglycemia [21]. Resistance training is a form of physical exercise that results in muscle hypertrophy, a direct increase in skeletal muscle mass. An increase in skeletal muscle mass results in the addition of muscle-related glucose transporters on the cell membrane and within it [22,23,24]. Thus, regular training ‘conditions’ the skeletal muscle to be primed and more adept to absorb blood glucose, along with being more insulin-sensitive [25,26].

The myokine myostatin (GDF-8) is a potent negative regulator of skeletal muscle growth that is upregulated in humans and animal models of obesity and in T1D patients, and downregulated following regular exercise [27,28,29,30,31]. Myostatin is primarily secreted by skeletal muscle and targets transcription factors involved in the promotion of muscle atrophy. This upregulation in T1D is linked with the muscle atrophy and loss of function seen in the disease, along with the uncontrolled hyperglycemia. The loss of myostatin (either genetically or pharmaceutically) is known to increase the number of glycolytic muscle fibers and overall muscle mass [32,33]. However, what remains poorly described is a comprehensive and integrative assessment of myostatin deletion within T1D, with an emphasis on its utilization as a potential pharmacological target in the therapeutic treatment of the disease, and this was the focus of this study. We hypothesized that myostatin deletion in a T1D mouse model would improve muscle function, maintain glucose homeostasis, preserve vascular function and improve overall metabolic function.

## 2. Results

Relevant baseline indices are included in Table 1, including body weight and whole body fat at the beginning (prior to the induction of diabetes) and end of the experimental study (day 28). The mass between the two groups did not differ significantly at baseline or the end of the study, and weight loss was similar between the two groups. WT mice had significantly more adiposity than myostatin KO mice, but neither group was altered significantly by T1D. The tissue measurements were obtained at the end of the experiment (day 28) and show a significantly increased gastrocnemius muscle in the myostatin KO mice. Further, myostatin KO mice exhibited no increase in liver and kidney weights, suggesting protection against the hepatomegaly (driven by hepatic lipidosis or glycogen overload) and renal hypertrophy that often afflict T1D patients [34,35,36,37].

### 2.1. Deletion of Myostatin Preserves Glucose Homeostasis in Type 1 Diabetes

**Figure 1:**
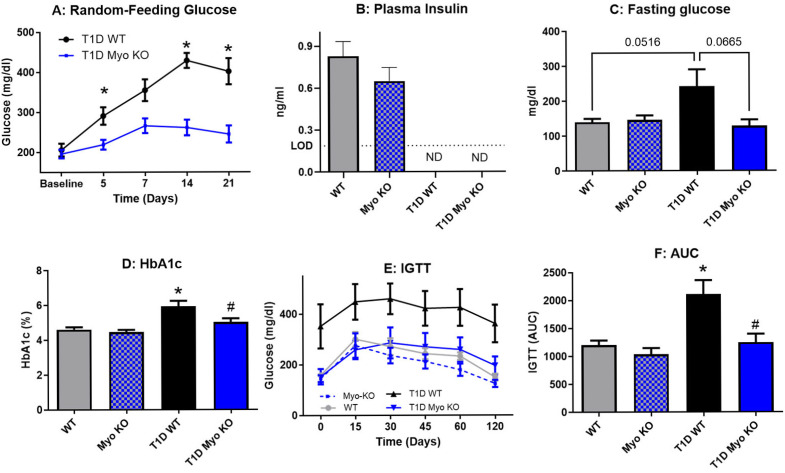
Deletion of myostatin preserves glucose homeostasis in Type 1 Diabetes. In panel (**A**), random-feeding glucose levels of the T1D Myo KO mice (blue line) are significantly decreased compared to the T1D WT controls over time. Panels (**B**–**E**) inform on glucose control at the end of the experiment (day 28), with STZ effectively lowering insulin to below levels of detection (LOD), and fasting glucose, HbA1c, and glucose clearance (IGTT) all being elevated in the T1D WT animals and lowered with myostatin deletion. Panel (**F**) represents the area under the curve (AUC) for panel (**E**). ND = not detectable. *n* was 8–10 for random-feeding glucose and was 4–5/group for IGTT. * = *p* ≤ 0.05 to WT. # = *p* ≤ 0.05 to T1D WT.

Glucose homeostasis was assessed throughout the study, with Figure 1A showing a steady increase in random-feeding glucose in T1D WT mice over the course of three weeks. The T1D Myo KO mice did not experience similar increases in hyperglycemia. At the end of the study, all mice in both groups treated with STZ had levels of insulin below detection (Figure 1B). Further, compared to T1D Myo KO mice, the T1D WT mice had elevated fasting glucose (Figure 1C) and impaired glucose clearance, as demonstrated by an intraperitoneal glucose tolerance test (IGTT) in Figure 1E. Myostatin deletion decreased the area under the curve of the IGTT to levels of control (Figure 1F). HbA1c, a relatively stable measure of glucose homeostasis as it is a 3-month average of glycosylated hemoglobin, was significantly elevated in the T1D WT mice. This highlights the significant impact that a sudden and sustained onset of hyperglycemia can have on systemic glucose regulation.

### 2.2. Deletion of Myostatin Preserves Muscle Function in Type 1 Diabetes

**Figure 2:**
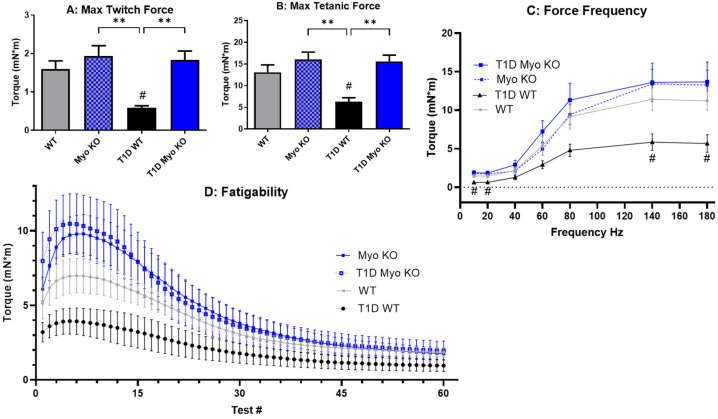
Deletion of myostatin preserves muscle function in Type 1 Diabetes. In panel (**A**), twitch force is shown to be unchanged across the myostatin groups compared to WT, but is significantly reduced in T1D WT. Isometric contraction is also impaired with T1D in WT mice, as shown at 100 Hz (**B**), and also lower stimulation, as seen in panel (**C**). Fatigability (**D**), while reduced in initial torque in the WT mice, is not significantly lower with T1D or reduced in rate or maximal fatigue. *n* = 5–6 per group. ** = *p* ≤ 0.01 as noted and # = *p* ≤ 0.05 to T1D WT.

The results of the in vivo plantarflexion examining muscle performance reveal that muscle function is significantly impaired with T1D in WT mice (Figure 2A–C). This deficit is observed in a muscle twitch (Figure 2A) as well as isometric contraction (Figure 2B,C) at low stimulation (10–20 Hz) and higher stimulation (100–180). However, myostatin deletion preserved maximum force generation even after diabetes was induced, as the T1D-Myo KO mice showed a preservation of function, particularly with regard to muscle torque production (Figure 2B,C). Fatigability was also tested (Figure 2D); however, there was no change in the rate of fatigue or lowered force generation between any of the groups. This is consistent with the literature, demonstrating that myostatin upregulates glycolytic fibers and diabetes preferentially targets glycolytic fibers (and, thus, neither would impact fatigability).

### 2.3. Deletion of Myostatin Protects Against Endothelial Dysfunction in Type 1 Diabetes

**Figure 3:**
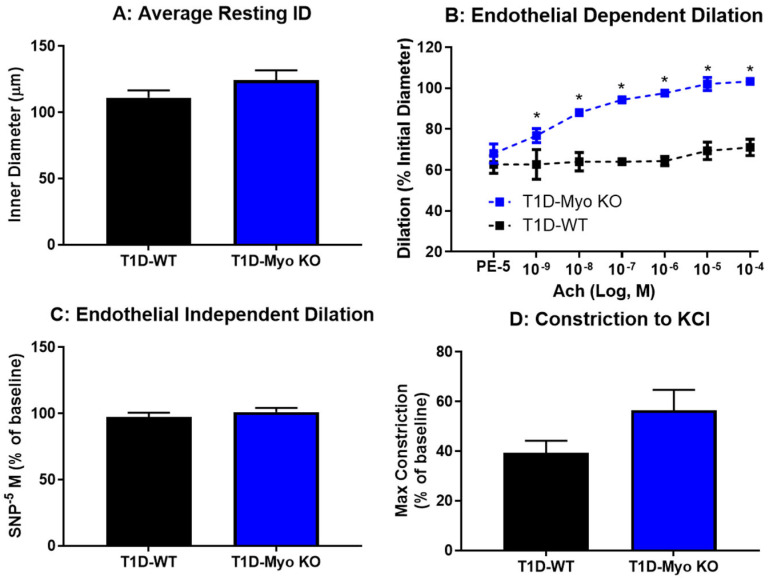
Deletion of myostatin protects against endothelial dysfunction in Type 1 Diabetes. Gracilis arterioles were isolated from each T1D group and, upon equilibration, showed no differences in resting diameter (**A**). Panel (**B**) shows significant endothelial dysfunction in T1D-WT mice, as assessed by decreased dilation to acetylcholine. Endothelial-independent dilation (**C**) to SNP was unchanged between the groups, as well as constriction to saturated KCl (**D**). *n* = 3–4 per group. * = *p* ≤ 0.05 compared to matched dose response.

Figure 3 evaluated vascular function in the skeletal muscle of the STZ groups using pressure myography. The overall resting diameter of the gracilis arterioles was similar between the groups (Figure 3A). Endothelial function was assessed (Figure 3B) using acetylcholine as an endothelial dependent dilator. T1D-WT arterioles showed significantly impaired endothelial function, with very little dilation to even the highest concentration of acetylcholine. However, the T1D-Myo KO mice displayed significant protection from endothelial dysfunction, with a significant dilation response to each dose. In light of the significant endothelial dysfunction accompanying diabetes in the control, endothelial-independent dilation was assessed (Figure 3C) using a bolus of sodium nitroprusside (SNP), a spontaneous nitric oxide donor. There was a robust and similar dilation to SNP in both groups, suggesting that smooth muscle cell function is still present in both groups and that the fibrosis that often occurs with long-term hyperglycemia has not fully presented. Finally, maximum constriction to saturated KCl (Figure 3D) showed no significant differences between overall constriction between the T1D groups. These results demonstrate that the STZ model substantially impairs vascular endothelial function in skeletal muscle, similar to humans experiencing poorly managed T1D, and that myostatin deletion can serve as a buffer to protect against vascular dysfunction, preserving overall cardiovascular and skeletal muscle function.

### 2.4. Deletion of Myostatin Protects Against Metabolic Dysfunction in Type 1 Diabetes

**Figure 4:**
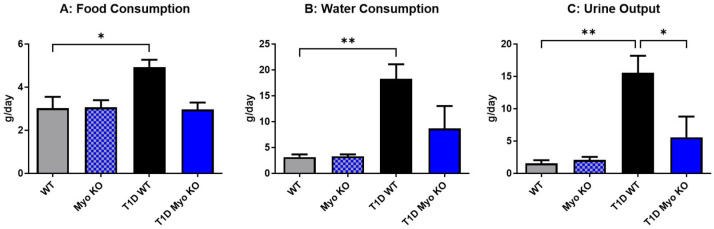
Deletion of myostatin protects against metabolic dysfunction in Type 1 Diabetes. Metabolic indices were assessed in all groups. Panel (**A**) shows a significantly increased food intake in the T1D-WT mice, whereas the T1D Myo KO mice remained satiated. Panel (**B**,**C**) show fluid dynamics in all groups, with the T1D-WT mice demonstrating significant polydipsia (**B**) and polyuria (**C**) compared to controls. In contrast, T1D Myo KO mice had improved fluid dynamics, remaining at the levels of the control groups. *n* = 5–6 per group. * = *p* ≤ 0.05 ** = *p* ≤ 0.01 against the illustrated groups.

Metabolic cages were used to assess food consumption and fluid dynamics in all groups. The results showed significant hyperphagia (Figure 4A), polydipsia (Figure 4B), and polyuria (Figure 4C) associated with T1D in the WT mice, mimicking the human population afflicted with uncontrolled hyperglycemia. Notably, myostatin deletion was effective at protecting all of these variables in T1D. These metabolic indices further reinforce the above data that glucose homeostasis is maintained with myostatin deletion, as it is the glycosuria in the urine that drives additional water loss and overall plasma and cellular dehydration in patients with hyperglycemia.

## 3. Discussion

Currently, T1D is an irreversible life-long disease that is driven by the destruction of pancreatic β-cells and removes the ability of an individual to produce insulin. From a therapeutic perspective, the maintenance of glucose homeostasis is key to a T1D patient’s long-term resistance to morbidity and mortality. As skeletal muscle is the largest glucose sink in most individuals, the overall amount (mass) and health (function) of this organ are vital components of glucose homeostasis. However, T1D patients are particularly susceptible to diabetic myopathy, a condition whereby they will experience atrophy and weakness of skeletal muscle [38,39]. The ability of skeletal muscle to appropriately maintain perfusion: demand matching is vital to the long-term health-span of any animal and directly impacts performance [40,41]. As such, it would be inherently valuable to find therapeutic targets that prevent the loss of skeletal mass and function in this vulnerable population.

This study utilized animals deficient in myostatin, a myokine well characterized as a potent negative regulator of skeletal muscle mass. Previous studies have identified a role for myostatin in T1D but our study sought to define its role in maintaining glucose homeostasis (independent of insulin) and protect against vascular dysfunction, with the specific goal of determining how these two variables influence the ability of skeletal muscle to respond to stimulation and also resist fatigue. We also quantified its ability to protect against the multitude of systemic and progressive pathologies that accompany T1D [42]. Type 1 diabetes was chemically induced (via STZ) in our mouse model and outcomes were monitored over 28 days, which is nearing the limit that the animals can survive without treatment of exogenous insulin. In our control mice, we observed a rapid and sustained presentation of hyperglycemia over the course of the study (Figure 1A), including impaired glucose clearance (Figure 1E). Insulin was determined to be reduced to undetectable levels in our T1D model (Figure 1B), and the inability of the animals to utilize carbohydrate metabolism resulted in whole-body weight loss (Table 1), despite the presentation of hyperphagia (Figure 4A). Further recapitulating the clinical phenotype of T1D patients was the observation of significant vascular endothelial dysfunction in skeletal muscle (Figure 3B). Taken together, our experimental outcomes were effective in capturing the most vital presentation of the disease. For the assessment of skeletal muscle function, we utilized in vivo plantarflexion for the purpose of examining the ability of skeletal muscle to generate torque, an integrative and foundational movement that informs on overall muscle function. The results showed that T1D significantly reduces muscle function (Figure 2A,B) and this deficit is significant at both low and high stimulation (Figure 2C). Interestingly, while T1D lowers overall muscle function, it did not appear to alter resistance to fatigue (Figure 2D), suggesting that the glycolytic fibers known for increasing muscle performance might be preferentially depleted in T1D.

Our initial results regarding the effect of myostatin deletion in the T1D model revealed a significant resistance to glucose dysregulation, which was quantified by lower plasma glucose, HbA1c, and glucose clearance. Notably, and despite Myo KO mice having substantially greater amounts of skeletal muscle, this did not translate to increased muscle function as baseline torque values were not significantly improved (Figure 2A–C). However, in our T1D model and independent of insulin, Myo KO mice were able to entirely resist the impairments in muscle function. Further supporting the positive role of augmented skeletal muscle mass driven by myostatin deletion was the significant improvement in endothelial function (Figure 3B) and improvement in fluid balance (Figure 4B,C). Indeed, the liver and kidney tissue weights were also significantly lower in the T1D Myo KO mice, suggesting a reduction in risk regarding the development of hepatomegaly and renal hypertrophy.

Taken together, the results of our study show that constitutive myostatin deletion shifts the hyperglycemic state of T1D mice to a more normoglycemic state and maintains muscle function and mass. These results identify several promising outcomes in looking towards the use of myostatin as an adjunctive therapy for T1D patients. Importantly, this study highlights several key points about skeletal muscle that we believe can drive future innovation in creating a healthier future for T1D patients. The downregulation of myostatin is known to upregulate glycolytic fibers and augment muscle mass and function [43,44]. Uniquely, it appears there is also an inverse relationship, with resistance training downregulating myostatin regardless of age or sex [45,46,47]. In theory, myostatin as a therapeutic could be targeted for amplification using this dual inverse relationship, with prescription exercise allowing individuals to partake in personalized resistance training in conjunction with an adjunct treatment of a myostatin inhibitor. This schema, in combination with low-dose insulin therapy, would combat the cardiometabolic effects of the disease and also subsequently slow the process of acquired insulin resistance associated with T1D [48]. This study builds on that foundation and expands on this research given the enhanced muscle function observed with myostatin deletion in T1D. Further credence is provided by the lack of an impact on fatigue resistance, which is largely controlled by oxidative and insulin-sensitive muscle fibers. Decreasing the circulating levels of this myokine promotes an increase or maintenance of existing skeletal muscle tissue, resulting in a larger concentration of insulin-insensitive tissue and serving as a buffer against catabolic diseases. While insulin-dependent GLUT transporters (particularly GLUT4) have been extensively studied, there are several key gaps in this field that remain poorly defined. It appears likely that skeletal muscle fiber type has a differential distribution of GLUTs and, as this study highlights, additional exploration into this field could prove advantageous in patients that have metabolic perturbations. This knowledge can be broadly leveraged in terms of identifying therapeutics (either preventative, pharmaceutical, or nutritional interventions) that preserve and protect glycolytic muscle fibers. Further, personalized prescription exercise regimens (e.g., resistance versus aerobic training) that enhance selective muscle fibers should be considered in populations that are susceptible to catabolic diseases that preferentially deplete specific skeletal muscle fibers, as has been observed in obesity and aging [49].

The implications from this study have several important limitations. The first is that the ability of an individual to exercise at a level that conveys benefit is limited not only by skeletal muscle mass and function but also by subjective feelings of energy and fatigue [50,51,52]. More importantly, while these overlapping concepts are separate and discrete variables, they have a significant impact on overall performance [53,54,55]. Indeed, type 1 diabetic patients are well documented for increased feelings of fatigue and/or a lack of energy independent of their glycemic state [56,57,58]. The presented study provides convincing evidence that myostatin deletion has the capability to preserve muscle function in a T1D rodent model. However, full clinical translation of the work will need to assess additional variables for a complete evaluation of overall enhancement of muscle performance. Within the study itself, several important observations must be noted as additional variables that could inform on the role of myostatin and systemic dysfunction induced by T1D. The first is the possibility that myostatin has a direct and unidentified role within the liver. Hence, myostatin inhibition could enhance liver function at baseline or prevent liver dysfunction (i.e., hepatic lipidosis and/or glycogen hepatopathy in T1D). Similarly, baseline improvements to mitochondrial function or skeletal muscle contraction with myostatin inhibition could also serve as a mechanism whereby the systemic dysfunction induced by T1D is avoided. Additionally, while myostatin has no acute effect on vascular function, a deleterious role with long-term exposure has not been disproved. Further, a longer-term project is not able to be conducted due to STZ-treated animals requiring exogenous insulin for survival. Another limitation is the use of only one sex (males) in the study. While clarity regarding the mechanism is needed, multiple studies confirm that the female sex has a higher distribution of oxidative fibers compared to the male sex, along with an increased resistance to fatigue [59,60,61]. As such, it is intriguing to speculate that a therapeutic treatment targeting glycolytic fibers (like myostatin) would be of greater benefit in the female sex, especially concurrent with T1D. Comparative studies utilizing fiber-type-specific therapeutics in skeletal muscle will need to closely account for sex differences.

Our study delivers a compelling rationale for targeting the myostatin pathway as a potential adjunct therapy for T1D patients, with the purpose of providing better control of glucose homeostasis, preserving skeletal muscle mass and function, and combating the deleterious cardiometabolic consequences that accompany this disease.

## 4. Materials and Methods

**Figure 5:**
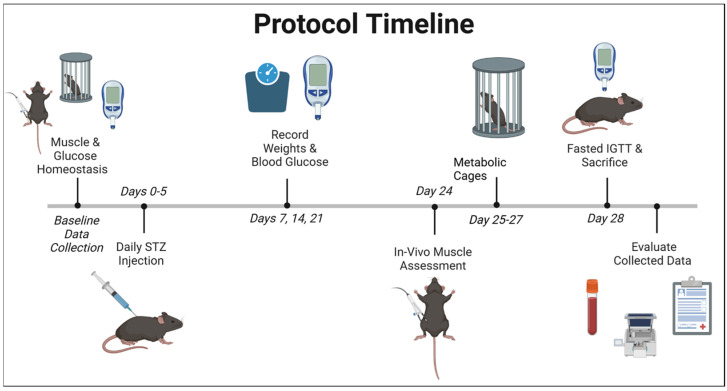
Schematic of protocol timeline: created in BioRender.

Animals and Supplies: Adult male mice between 12 and 30 weeks of age were used for the duration of the study. Mice with constitutively deleted myostatin on the C57/Bl6J background were utilized, as described previously [62,63]. Overall, the total number of mice per group was 10–12, and experimental numbers are listed within the figures themselves, if different from total experimental numbers. Every effort was made to utilize a study design that emphasized non-invasive experiments, and where possible, a baseline prior to STZ injection was used as a control to minimize animal use. For ease of review, the nomenclature of the groups includes wild type (WT), myostatin deletion controls (Myo KO), and the induction of T1D using Streptozotocin (STZ) in both groups (T1D-WT and T1D-Myo KO). For the induction of T1D, mice were injected (IP) with a low-dose of STZ, being administered at 50 mg/kg for five consecutive days [54]. Mice undergoing STZ injection were monitored over 28 days before the termination of the experiment. Insulin was assessed using the ALPCO 80-INSMS-E01 to determine insulin levels in the mice. The chemicals were purchased from Millipore Sigma, unless otherwise noted. The overall experimental timeline is presented in Figure 5 and labeled Protocol Timeline.

Assessment of Glucose Homeostasis: Blood was obtained via a tail snip or subsequent to sacrifice via a cardiac puncture, and glucose variables were assessed using Medline’s Evencare G2 glucometer and PTS Diagnostics A1CNow + analyzer. Glucose clearance was assessed using an intraperitoneal (IP) glucose tolerance test (IGTT). Mice underwent a 12 h fast prior to IGTT, weighed, and then injected with 2g of dextrose/kg body weight. Blood glucose levels were obtained at baseline (0) and 15, 30, 45, 60 and 120 min using the tail stick method.

Assessment of Whole-Body Adiposity: A Minispec Body Composition Analyzer (Bruker in Billerica, Massachusetts, USA; Model no. LF90II) was utilized to assess body fat percentage.

Assessment of Metabolism: Metabolic cages were utilized to assess food and water intake and urine output. Mice were placed into metabolic cages for 48 h and allowed to acclimate for the first 24 h, with the data recorded and analyzed for the last 24 h.

Assessment of Muscle Function: Muscle performance was measured using in vivo plantarflexion with Aurora Scientific’s 1300 A Whole Animal System with electrical stimulation of the gastrocnemius muscle. In brief, mice were anesthetized and maintained with isoflurane during the muscle assessment. Hair distal to the hip was removed using a depilatory. Mice were placed on a heated pad and the knee secured. The foot was aligned on the foot plate and, upon toe flexion, secured with surgical tape. Electrodes were placed subcutaneously on the medial aspect of the gastrocnemius for stimulation of the muscle. An isometric contraction at 100 Hz was utilized to ensure appropriate placement and security of the limb. Subsequently, the limb underwent various stimulations, including twitch, a force frequency protocol at 10, 20, 40, 60, 80, 140, and 180 Hz (with 3 min between each frequency), and a fatigue protocol at 60 Hz for every 5 s for 5 min. The mice were then recovered with a heating pad and flow-by oxygen until conscious.

Assessment of Vascular Function: The gracilis arteriole was excised upon sacrifice and placed in ice-cold Krebs solution, as previously published [64,65,66]. The Krebs solution was composed of 118 mM NaCl, 25 mM NaHCO_3_, 1.2 mM MgSO_4_, 11 mM D-glucose, 4.7 mM KCl, 2.5 mM CaCl_2_, and 1.2 mM KH_2_PO_4_. The arteriole was cannulated on glass pipettes using 10-0 silk on a Living Systems (LS) small vessel arteriograph, heated to 37 °C, and pressurized to 60 mmHg using a Pressure Servo Controller (Living Systems PS-200-S). The vessels were allowed to equilibrate for 30 min before undergoing reactivity curves. The inner diameter of the vessels were monitored on an Accu-Scope EXI-310 using Colorado Video 307 digital calipers. Upon the development of spontaneous tone, the vessels were evaluated for endothelial and smooth muscle cell function. For the evaluation of endothelial dependent dilation, the vessels were preconstricted with phenylephrine (10^−5^ M PE) and then underwent a dose response to acetylcholine (10^−9^–10^−4^ M Ach). Vessels that did not respond to phenylephrine were excluded from the study. Upon washout of acetylcholine, endothelial-independent dilation was assessed using a bolus of sodium nitroprusside (10^−5^ M SNP), subsequent to preconstriction with phenylephrine, as described above. After pharmacological stimuli, vessels were washed and maximally constricted with saturated KCl to determine the overall patency of the smooth muscle.

Statistical Analysis: Statistical analysis was performed using the GraphPad Prism 10.4.1 software. All data are presented as mean ± SEM and significance tested using an unpaired Student *T*-test, one-way ANOVA with Tukey’s multiple comparison or two-way ANOVA repeated measures, where appropriate. For all analysis, statistical significance is noted at *p* ≤ 0.05.

## Figures and Tables

**Table 1:** Body composition at baseline and after T1D (at 28 days) of the groups is included in Table 1. Data shown as mean ± SEM. *n* = 10–12/group. ^ = *p* ≤ 0.05 to WT and * = *p* ≤ 0.05 to T1D-WT.

Body Composition	WT	KO	WT	KO
	Baseline		T1D (Day 28)	
Weight (g)	38.7 ± 1.0	39.5 ± 0.8	33.9 ± 0.6	35.3 ± 0.7
Weight Loss (%)			12.1 ± 1.5	10.5 ± 1.5
Fat (%)	13.9 ± 2.4	3.2 ± 1.8 ^	12.0 ± 1.6	3.2 ± 1.7 *
	**Organ Weights (mg)**			
Liver			1282 ± 64.8	1050 ± 55.8 *
Gastroc			187.0 ± 6.1	293.2 ± 15.3 *
Heart (wet wt.)			164.4 ± 8.0	169.4 ± 8.0
Kidney			228.8 ± 6.7	202.9 ± 6.5 *

## Data Availability

The data that support the findings of this study are available upon reasonable request from the corresponding author.

## Abbreviations

The following abbreviations are used in this manuscript:

T1D	Type 1 Diabetes Mellitus
STZ	Streptozotocin
Myo	Myostatin
